# Normal RNAi response in human fragile × fibroblasts

**DOI:** 10.1186/1756-0500-2-177

**Published:** 2009-09-09

**Authors:** Charlotte Madsen, Karen Grønskov, Karen Brøndum-Nielsen, Thomas G Jensen

**Affiliations:** 1 The Kennedy Center, Gl. Landevej 7, 2600 Glostrup, Denmark; 2 Institute of Human Genetics, University of Aarhus, 8000 Aarhus C, Denmark; 3 Faculty of Health Sciences, University of Copenhagen, Denmark

## Abstract

**Background:**

Fragile × syndrome is caused by loss of expression of the FMRP protein involved in the control of a large number of mRNA targets. The Drosophila ortholog dFXR interacts with a protein complex that includes Argonaute2, an essential component of the RNA-induced silencing complex (RISC). Furthermore dFXR associates with Dicer, another essential processing enzyme of the RNAi pathway. Both microRNA and microRNA precursors can co-immunoprecipitate with dFXR. Consequently it has been suggested that the Fragile × syndrome may be due to a defect in an RNAi-related apparatus.

**Findings:**

We have investigated the RNAi response in Fragile × patient cells lacking FMRP compared with normal controls. RNAi responses were successfully detected, but no statistically significant difference between the response in normal cells compared to patients cells was found - neither one nor two days after transfection.

**Conclusion:**

Our data show that in human fibroblasts from Fragile × patients lacking FMRP the RNAi response is not significantly impaired.

## Findings

Fragile × syndrome is caused by loss of expression of the FMRP protein involved in the control of a large number of mRNA targets [1]. The Drosophila ortholog dFXR interacts with a protein complex that includes Argonaute2, an essential component of the RNA-induced silencing complex (RISC) [2]. Furthermore dFXR associates with Dicer, another essential processing enzyme of the RNAi pathway [3]. Both microRNA and microRNA precursors can co-immunoprecipitate with dFXR [3], and it was shown that FMRP can act as a miRNA acceptor protein for the ribonuclease Dicer and facilitate the assembly of miRNAs on specific target RNA sequences [4,5].

Consequently, it has been suggested that the Fragile × syndrome may be due to a defect in an RNAi-related apparatus [6] although in Drosophila S2 cells dFXR is not required for an RNAi response [7]. RNAi is a naturally occurring mechanism of gene regulation that induces sequence-specific knock-down of gene expression at the post-transcriptional level [8]. Regulation of gene expression by RNAi utilizes endogenous cellular pathways in which double-stranded RNA molecules produced from endogenous or foreign DNA are processed into short double-stranded RNA molecules of 21-23 nucleotides. These small interfering RNA (siRNA) molecules are incorporated into RISC that facilitates degradation of the target.

Recently the RISC function was analysed in mouse embryo fibroblasts (MEFs) from FMRP wildtype or knockout (FMR1-/-) littermates [9]. Here it was shown that cells lacking FMRP have normal RISC activity, since FMRP and RISC were associated with distinct pools of mRNAs.

**Figure 1:**
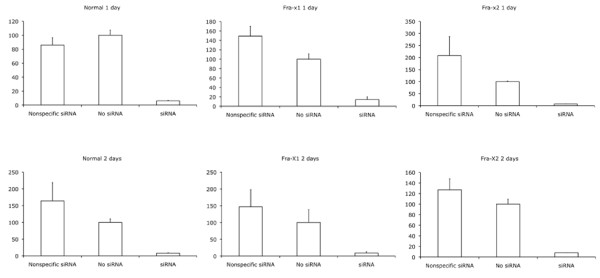
**Relative luciferase expression showing the RNAi response in human fibroblasts**. Primary human skin fibroblasts from two fragile × patients (Fra-X1 and 2) and a normal control person with a normal karyotype were transfected with the plasmid pGL3-basic (Promega) encoding luciferase and siRNA against the luciferase (GL3 siRNA, MWG-Biotech). GL3 siRNA targets the luciferase sequence 5'-CUUACGCUGAGUACUUCGATT -3' [11]. As control for the transfection frequency, cells were also transfected with the plasmid pCMVβ (Clontech) encoding beta-galactosidase. Luciferase and beta-galactosidase activities were measured one and two days after transfection using kits from Pierce and Promega, respectively. Transfections were performed in triplicates using X-tremeGeNE siRNA Transfection Reagent as suggested by the manufacturer (Roche). Subconfluent cells in 25 cm^2 ^tissue culture flasks were transfected with a mixture of 100 μl transfection reagent diluted in Opti-MEM together with 6 μg of each of the reporter plasmids and 2 μg of the siRNA oligonucleotides. The primary fibroblasts were obtained from 2 patients with a trinucleotide expansion leading to a full mutation in the FMR1 gene, and, thus, no FMRP protein detectable using western blotting (data not shown). A negative control siRNA with no significant homology to any known gene sequences from mouse, rat, or human was also purchased from MWG-Biotech. Shown on the Y-axes are the luciferase expression divided by the beta-galactosidase expression, setting the ratio to 100 for cells transfected without siRNA. Error bars: Standard deviation. The experiments comply with the National regulations on cells from approved biobanks. The primary human fibroblasts were cultured according to standard procedures and used in early passages.

We have investigated the RNAi response in Fragile × patient cells lacking FMRP compared to fibroblasts from normal individuals. Primary fibroblasts from patients and controls were co-transfected with a luciferase plasmid and siRNA oligonucleotides against the luciferase gene. As control for the transfection frequency, cells were also transfected with the plasmid lacZ encoding the beta-galactosidase. As seen in Fig 1 RNAi responses were successfully detected, but no statistically significant difference between the response in normal cells compared to patients cells was found - neither one nor two days after transfection (two-tailed T-test). However, we cannot rule out that the short term kinetics of the RNAi reponse in normal and patient cells differ.

## Conclusion

Our data support the conclusions made by Didiot et al. that cells lacking FMRP have a normal RNAi response. Since FMRP together with the autosomal paralogs FXR1P and FXR2P constitutes a family of RNA bindings proteins, it is conceivable that functional redundance exists among these, contributing to the observed results [10]. Further experiments are required to analyse the involvement of FMRP in the miRNA pathway in human cells.

## Competing interests

The authors declare that they have no competing interests.

## Authors' contributions

CM performed all experiments supervised by KG and TGJ. KBN and TGJ participated in experimental designs and finalised the manuscript. TGJ drafted the manuscript along with KBN and KG. All authors read and approved the final manuscript.
